# Digital therapeutic for hypertension improves physician-patient communication and clinical inertia: a survey of physicians who implemented CureApp HT in clinical practice

**DOI:** 10.1038/s41440-024-01899-x

**Published:** 2024-10-12

**Authors:** Tomohiro Katsuya, Fumi Hisaki, Mitsuharu Aga, Yumi Hirayama, Yusuke Takagi, Yuko Ichikihara, Tomoyuki Tanigawa

**Affiliations:** 1 Katsuya Clinic, Amagasaki, Japan; 2 Department of Clinical Gene Therapy, Osaka University Graduate School of Medicine, Osaka, Japan; 3 Medical Division, CureApp Inc., Tokyo, Japan; 4 Teikyo Academic Research Center, Tokyo, Japan; 5 St. Luke’s International Hospital, Tokyo, Japan

**Keywords:** Clinical inertia, Digital therapeutics, Hypertension, Physician-patient communication, Transtheoretical model

## Abstract

In the 2019 Guidelines for the Management of Hypertension by the Japanese Society for Hypertension, lifestyle modification is recommended for all individuals except those with normal blood pressure. However, no detailed methods have been established to achieve the target blood pressure and resolve clinical inertia. CureApp HT, a digital therapeutic for hypertension that contributes to blood pressure reduction through lifestyle modification, was approved as software as a medical device for reimbursement by Japanese national health insurance in September 2022. This study aimed to survey physicians who implemented CureApp HT to assess how it changes physician-patient communication and contributes to clinical inertia resolution. A questionnaire survey was conducted at three time points: before the first prescription (first survey), 3 months (second survey), and 6 months (third survey) after the first prescription for physicians who had implemented CureApp HT. The primary outcome was the total score of five items on a Likert scale related to physician-patient communication, and it was analyzed based on the 47 physicians who responded to all three questionnaires. The total score of physician-patient communication significantly improved after 6 months of the introduction of CureApp HT, reflecting that physicians observed positive changes in patients’ knowledge and attitudes regarding hypertension treatment. Furthermore, the number of physicians who set a target home blood pressure of 125/75 mmHg for their patients significantly increased. CureApp HT allows physicians to recognize changes in patients’ disease knowledge and treatment attitudes, enabling them to set more stringent blood pressure targets and addressing clinical inertia.

**Figure:**
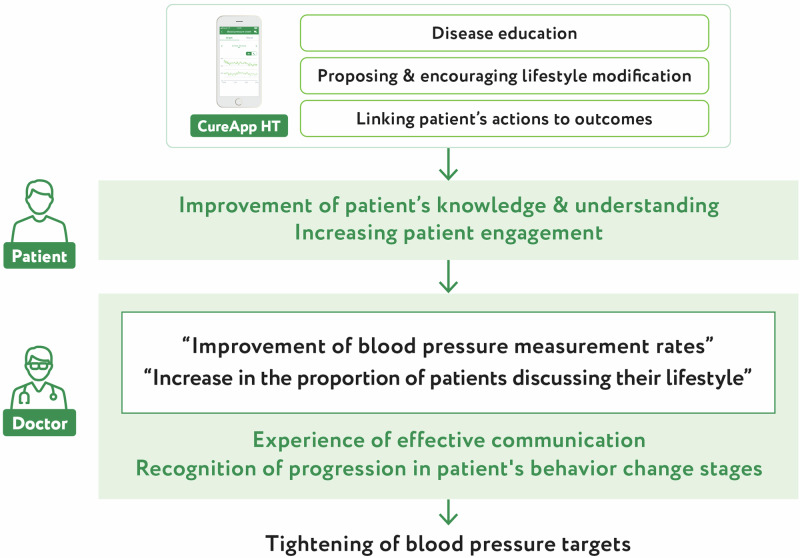
Physicians who implemented CureApp HT recognized changes in the patients’ stages of behavioral change through improvements in patients’ knowledge of the disease and their attitudes towards treatment, and by experiencing more effective communication, they set stricter blood pressure targets.

Physicians who implemented CureApp HT recognized changes in the patients’ stages of behavioral change through improvements in patients’ knowledge of the disease and their attitudes towards treatment, and by experiencing more effective communication, they set stricter blood pressure targets.

## Introduction

Hypertension is one of the most prevalent diseases in Japan, affecting approximately 43 million individuals [1]. Essential hypertension is estimated to account for 90% of all hypertension cases and is a primary risk factor for cerebrovascular and cardiovascular events [1]. Treatment for essential hypertension involves pharmacotherapy and lifestyle modification. The Guidelines for the Management of Hypertension 2019 by the Japanese Society for Hypertension (JSH) recommend lifestyle improvements for every patient, except those with normal blood pressure [1], and set goals for each lifestyle category. However, there is currently no established methodology that provides detailed guidance for each clinical setting. Considering the diverse backgrounds of patients and the limited time available for outpatient visits, it is challenging to provide effective, individualized guidance. Although improvements in outcomes through multidisciplinary involvement have been explored [2], obtaining such resources in individual clinical settings is highly challenging.

To address these issues, CureApp HT, a digital therapy for hypertension, was developed. This software as a medical device consists of a patient application installed on a smartphone and a web application for physicians and medical practitioners. Patients acquire knowledge about the disease and practice lifestyle modifications according to the application guidelines. They can choose from specific lifestyle improvement actions suggested by the application and reflect on the relationship between the achieved blood pressure reduction and lifestyle improvement made thus far. The web application for physicians supports lifestyle guidance by displaying the background of blood pressure trends and lifestyle characteristics obtained from the patient application. Consequently, improvements in two-way communication can be expected.

In a randomized controlled trial (HERB-DH1 study) of CureApp HT for patients with essential hypertension who were not taking antihypertensive drugs, significant reductions were observed in the morning and evening home blood pressure, 24-h average blood pressure as measured by ambulatory blood pressure monitoring, and office blood pressure [3]. In addition, significant decreases were observed in salt check sheet score [4] and body weight. Based on this pivotal trial, CureApp HT gained regulatory approval in September 2022 as the world’s first digital therapy for essential hypertension.

Although the antihypertensive effect of CureApp HT has been confirmed in the HERB-DH1 trial and other reports [5], its impact on clinical practice experience and the physician-patient relationship is unknown. Hypertension treatment is associated with issues, such as difficulty in lifestyle modification, poor medication adherence, and clinical inertia. Clinical inertia is considered one of the major causes of insufficient treatment of diseases without subjective symptoms [6], and various factors related to medical providers, patients, and the healthcare system have been identified [7]. Clinical inertia in hypertension treatment is defined as “not starting treatment despite patients’ high blood pressure, or observing without intensifying treatment despite not achieving the target blood pressure indicated in the guidelines” [1]. It is worth investigating whether CureApp HT improves not only blood pressure but also clinical practice experiences, including physician-patient communication, which could lead to a solution to clinical inertia.

Therefore, this study aimed to survey physicians who implemented CureApp HT to investigate changes in their clinical practice experiences and examine how its implementation in clinical practice contributes to resolving clinical inertia.

## Methods

### Study design

This study involved a questionnaire survey of physicians who prescribed CureApp HT for the first time. At three points in time—before the first prescription of the application and three and six months after prescribing—the participating physicians answered questions sent via Google Forms. This study was conducted in accordance with the Declaration of Helsinki and Human Ethics Guidelines and approved by the Kitamachi Clinic Ethics Committee in Tokyo. Informed consent was obtained from all participants before study participation. In this study, participating physicians needed to have experience in the treatment of essential hypertension and be practicing in a facility that meets the requirements for insurance application for CureApp HT. Physicians who had previously used the CureApp HT in clinical trials or other settings were excluded.

### Survey items

Using the questionnaire (Table 1), we obtained responses to 13 questions regarding the following: length of consultation time for patients with hypertension, time spent speaking by physicians and patients during the consultation, contents of the conversation, frequency of patients’ blood pressure measurements, target blood pressure, whether the physician shared the treatment objectives, and the physician’s evaluation of communication with patients with hypertension (in the second and third questionnaire surveys, this evaluation focused on patients using CureApp HT). Moreover, we collected data on the location and type of medical institution, age of the physician, years of experience in treating hypertension, number of prescriptions for CureApp HT, and home blood pressure of the patients prescribed CureApp HT. Missing data were excluded.

**Table 1:** Survey questions

1. On average, how much time do you spend during the initial consultation for patients with hypertension (using CureApp HT)*?
2. On average, how much time do you spend during the follow-up consultations for patients with hypertension (using CureApp HT)*?
3. What is the ratio between the time the patient with hypertension (using CureApp HT)* talks and the time the doctor talks? (e.g., Check “4” if the ratio is 40% for the patient/60% for the doctor)
4. When the patient’s total speech is considered 100%, what percentage do they discuss the following topics? - Health condition - Lifestyle - Blood pressure readings - Medication - Others
5. When the doctor’s total speech is considered 100%, what percentage do you discuss the following topics? - Health condition - Lifestyle - Blood pressure readings - Medication - Others
6. What proportion of patients with hypertension (using CureApp HT)* are recording their home blood pressure?
7. When providing feedback on home blood pressure recordings to patients with hypertension (using CureApp HT)*, please indicate the frequency for each topic: - Difference from hypertension standards - Difference from shared target value - Measurement conditions - Antihypertensive medication - Others (1: Rarely – 5: Often)
8. Regarding the “target blood pressure” shared with patients with hypertension (using CureApp HT)*, which is closest? (Select one) - Below current home blood pressure of 135 mmHg or more - Home blood pressure goal of 135/85 mmHg - Home blood pressure goal of 125/75 mmHg - No specific shared target value - Others
9. Do you feel that you are able to share the “purpose of hypertension treatment” with patients with hypertension (using CureApp HT)*? (Select one) - Not shared - Partially shared - Generally shared - Clearly shared
10. If you answered “Not shared” or “Partially shared,” please tell us the reason. - Not understood - Lack of time to explain - Do not see the necessity - Others
11. Regarding your experiences in treating patients with hypertension (using CureApp HT)*, please let us know if you feel the following: (Always - Never, or “Don’t know”) - Feeling that the patient is proactive in treatment - Recognizing that the patient’s questions are appropriate - Having meaningful communication with the patient - Providing sufficient information to the patient - Feeling that the patient understands the provided explanation
12. Do you have the “2019 Guidelines for the Management of Hypertension” by the Japanese Society of Hypertension?
13. Please indicate what closely describes your current hypertension treatment practices (multiple options possible) - Follow the 2019 Guidelines - Check the 2019 Guidelines when unsure - Use the 2019 Guidelines to explain to patients - Refer to the 2019 Guidelines based on patient needs - Treat based on personal experience - Treat based on patient preferences

* for second & third survery

### Statistical analysis

The primary outcome was the total score of the five items on the Likert scale (Question 11 in Table 1, hereafter referred to as “physician-patient communication score”) related to physician-patient communication. We examined the change in this score from before the first prescription of CureApp HT. The items constituting the physician-patient communication score were “feeling that the patient is proactive in treatment,” “recognizing that the patient’s questions are appropriate,” “having meaningful communication with the patient,” “providing sufficient information to the patient,” and “feeling that the patient understands the provided explanation.” The evaluation based on the Likert scale was designed such that the score increases with positive changes, with 1, 2, 3, and 4 points for “never,” “rarely,” “sometimes,” and “always,” respectively, and the total score was the sum of these. If the answer to the question was “I don’t know,” the total score was calculated by multiplying the scores obtained from other questions by (5/number of questions with an answer other than “I don’t know”). If the answer to all questions was “I don’t know,” that response was not considered a valid response. Data from physicians who responded to all three questionnaires were included. Paired *t*-tests were used to evaluate the significance of changes in continuous variables, and chi-square or Wilcoxon signed-rank tests were employed to investigate changes in categorical outcomes. Primary analysis was performed using a null hypothesis value of 0. Additionally, for subgroup analysis, we conducted an analysis of covariance (ANCOVA) with covariates including physician age (divided into two groups: under 50 years or over), geographical region (Kanto region vs. other), years of experience in hypertension treatment (under 10 years or over), type of medical institution (clinic vs. hospital), possession of hypertension treatment guidelines (yes vs. no), and length of consultation for follow-up patients (0–5, 5–15, or >15 min). Considering the exploratory nature of this study, we did not adjust for multiplicity and set the two-sided significance level at 0.05.

## Results

### Characteristics of participants

From November 2022 to the end of June 2024, 152 physicians consented to participate in this study, and 149 physicians were enrolled after excluding three who did not meet the eligibility criteria. Among the 115 physicians who responded to the first survey, 77 actually prescribed CureApp HT. The second and third survey responses were obtained from 57 and 51 physicians, respectively. Most respondents were in their 40 s, comprising 46 (40%) of the total respondents (Table 2). More than half of the respondents (68%) had 15 or more years of experience in hypertension treatment, and 18% had 10–15 years of experience. Regarding the type of medical institution, 113 physicians (98%) were affiliated with clinics.

**Table 2:** Background of participating physicians

Characteristic	*N*	Percentage
Age (years)		
30–39	12	10.4
40–49	46	40.0
50–59	35	30.4
60–69	18	15.7
70–79	4	3.5
Geographical Location		
Hokkaido	2	1.7
Tohoku	3	2.6
Kanto	47	40.9
Chubu	10	8.7
Kansai	10	8.7
Chugoku	7	6.1
Shikoku	2	1.7
Kyushu & Okinawa	34	29.6
Duration of Hypertension Treatment (years)		
0–5	5	4.4
5–10	11	9.6
10–15	21	18.3
>15	78	67.8
Type of Medical Facility		
Clinic	113	98.3
Hospital	2	1.7

### Primary outcome

**Fig. 1:**
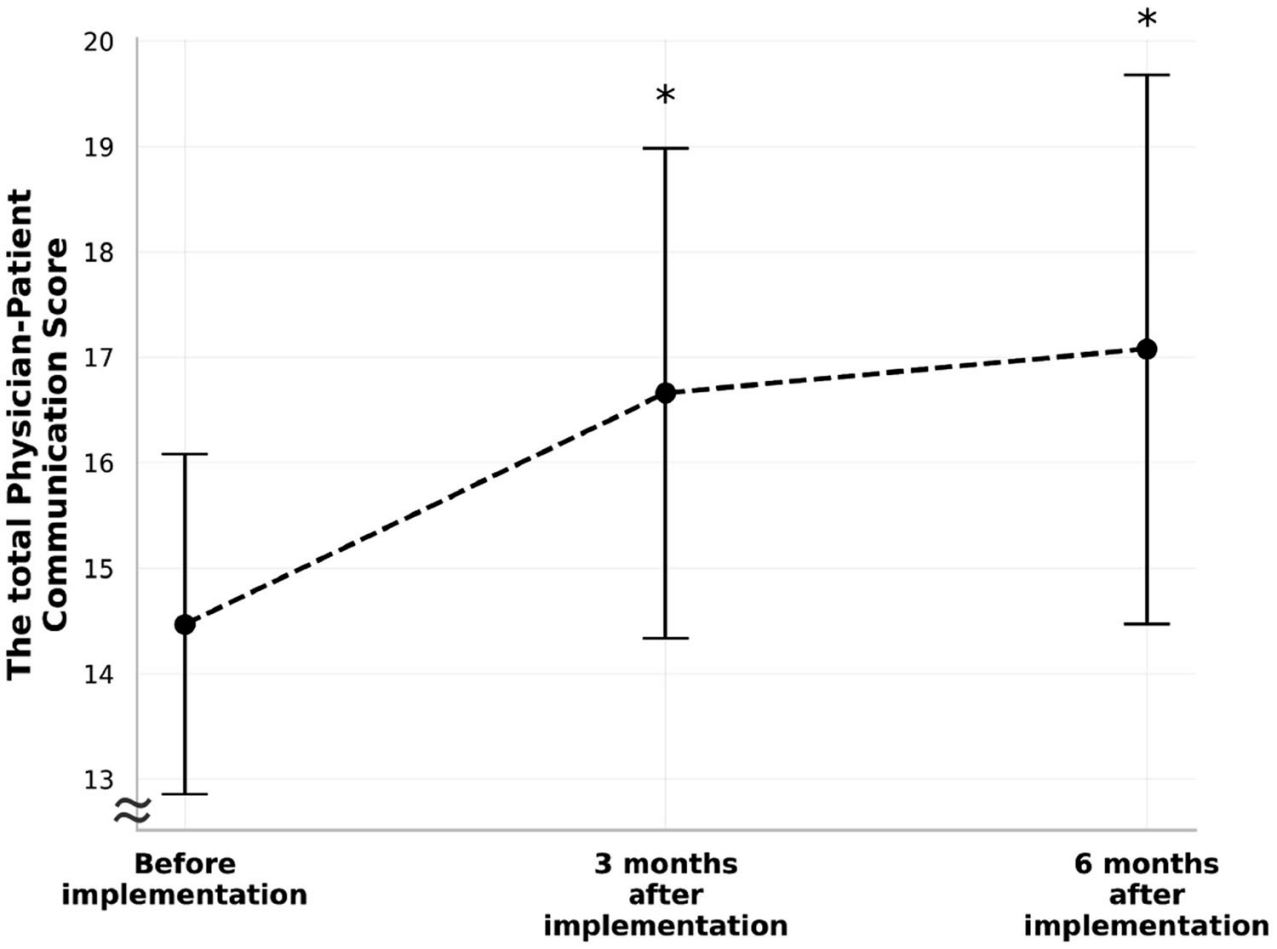
The total physician-patient communication score at the baseline and after 3 and 6 months of implementing CureApp HT (error bars represent standard deviation). *p < 0.05

**Fig. 2:**
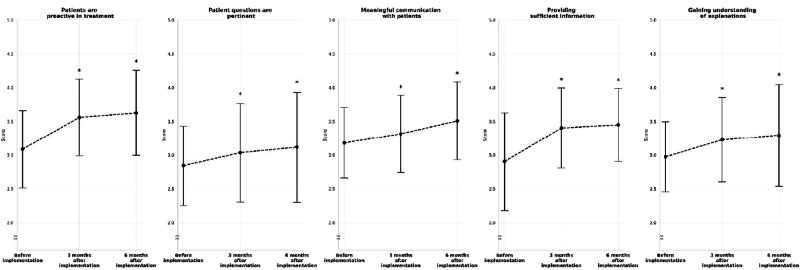
Physician-patient communication scores at the baseline and after 3 and 6 months of implementing CureApp HT (error bars represent standard deviation). *p < 0.05

The average total physician-patient communication score among the 47 physicians who responded to all three questionnaires was 14.5 (standard deviation [SD] 1.6) before the first prescription of CureApp HT, 16.7 (SD 2.3) 3 months after the first prescription, and 17.1 (SD 2.6) 6 months after the first prescription. The average change in score compared with baseline was +2.2 and +2.6 points at 3 and 6 months after the first prescription, respectively, showing a significant increase at both time points (*P* < 0.001 and *P* < 0.001, respectively) (Fig. 1). This trend was consistent for all five questions that were used to calculate the physician-patient communication score (Fig. 2).

### Subgroup analysis

According to the ANCOVA results, demographic variables of variables regarding health care delivery such as the physician’s age (50 years or older, effect size −0.30, 95% confidence interval [CI] −2.32 to 1.71, *P* = 0.76), geographical region (Kanto, effect size 1.24, 95% CI −0.91 to 3.39, *P* = 0.25), professional experience (more than 10 years, effect size 1.95, 95% CI −0.89 to 4.79, *P* = 0.17), type of facility (hospital, effect size 1.29, 95% CI −5.36 to 7.93, *P* = 0.70), possession of guidelines (yes, effect size 2.01, 95% CI −1.01 to 5.03, *P* = 0.19), and baseline length of consultation time (effect size −1.49, 95% CI −3.04 to 0.06, *P* = 0.06) did not have a significant impact on the communication score.

### Treatment goals

**Fig. 3:**
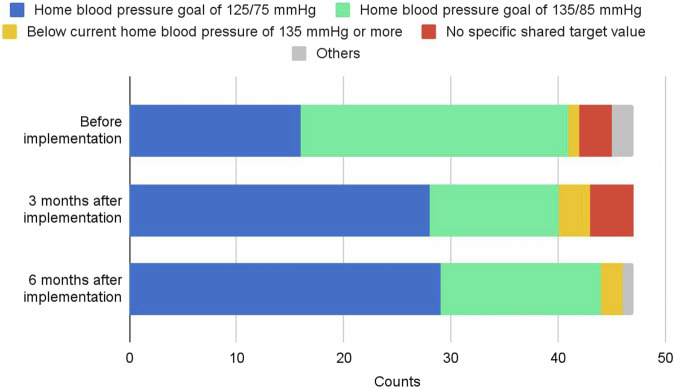
The proportion of home blood pressure targets presented to patients during the follow-up periods at the baseline and 3 and 6 months post-implementation of CureApp HT

The percentage of physicians who set a home blood pressure target of 125/75 mmHg (according to the JSH guidelines) was 34.0% before the first prescription of CureApp HT, and 59.6% and 61.7% at 3 and 6 months after the first prescription, respectively, showing a significant increase (*P* = 0.02 and *P* = 0.01, respectively) after implementation (Fig. 3). The percentage of physicians who could share the purpose of hypertension treatment with patients (“definitely” or “mostly”) was 83.0% before the first prescription of CureApp HT, and 95.7% and 89.3% at 3 and 6 months after the first prescription, respectively, indicating a significant increase (*P* = 0.001 and *P* = 0.03, respectively) after the prescription.

### Length of consultation time (initial visit)

The time spent seeing patients face-to-face at the initial visit was as follows: before the first prescription of CureApp HT: 0–5 min 2.1%, 5–10 min 27.7%, and > 10 min 70.2%; 3 months later: 0–5 min 2.1%, 5–10 min 23.4%, and >10 min 74.5%; and 6 months later: 0–5 min 6.4%, 5–10 min 34.0%, and >10 min 59.6% (Supplementary Fig. 1).

### Length of consultation time (return visit)

The time spent seeing patients face-to-face at the return visit was as follows: before the first prescription of CureApp HT: 0–5 min 48.9%; 5–10 min 44.7%, and >10 min 6.4%; 3 months later: 0–5 min 25.5%, 5–10 min 57.4%, and >10 min17.0%; 6 months later: 0–5 min 38.3%, 5–10 min 46.8%, and >10 min 14.9% (Supplementary Fig. 2).

### Percentage of patients recording home blood pressure

The percentage of patients who recorded home blood pressure regularly was 66.3% before the first prescription of CureApp HT, and 90.2% and 89.8% at 3 and 6 months after the first prescription, respectively, showing a significant increase (*P* < 0.001 and *P* < 0.001, respectively) after the prescription.

### Patient’s speech content

**Fig. 4:**
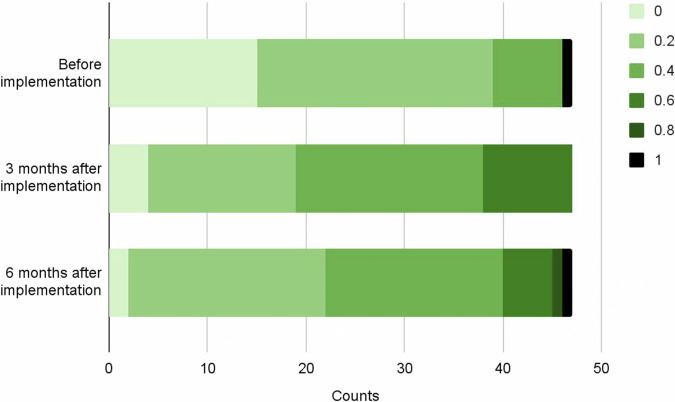
The proportion of topics (lifestyle habits) discussed by patients at the baseline and after 3 and 6 months of implementing CureApp HT

The median percentage of time patients spoke about their lifestyle during revisits was 20% before the first prescription of CureApp HT, and 40% at both 3 and 6 months after the first prescription. There was a significant increase at 3 months (*P* < 0.001) and 6 months *(P* < 0.001) after the first prescription (Fig. 4). No significant changes were observed in the speaking time regarding health status, blood pressure measurements, drug therapy, or other topics (Supplementary Fig. 3).

### Physician’s speech content

The proportion of time that the physician spoke about health status, lifestyle habits, blood pressure measurements, drug therapy, and other topics during revisits was almost consistent across the three time points (Supplementary Fig. 4).

## Discussion

This study found that physicians who implemented CureApp HT in their clinical practice experienced effective physician-patient communication. Specifically, they were able to provide patients with sufficient information, felt that the patients understood their disease well, and observed that the patients were actively engaged in their treatment, leading physicians to believe that they were able to communicate effectively. Furthermore, the amount of time spent discussing lifestyle habits during consultations increased, and the target blood pressure values given by the physicians became more in line with the guidelines. The possible reasons why physicians who prescribed CureApp HT experienced more effective communication during consultations were changes in the patient’s attitude towards treatment. This study indicated that these changes involved an increase in spontaneous patient talk about “lifestyle habits” and an increase in the rate of blood pressure measurement at home.

In the HERB-DH1 study, a pivotal clinical trial of CureApp HT showed that the more patients used the application to record their lifestyle improvement actions, the more successful they were in lowering their blood pressure [8]. These findings suggest that patient actions and the actual lowering of blood pressure mutually promote each other and that the improvement of self-efficacy plays an important role in this mechanism [9]. Another study found that self-efficacy was related to adherence to self-care. Moreover, a gap exists in perception between physicians and patients regarding hypertension management, which hinders effective communication; for example, patients reported not hearing what the physician said, even though the physician believed they had effectively communicated [10]. In this study, this gap appeared to be bridged by the improved knowledge and positive attitudes of patients facilitated by the use of CureApp HT, resulting in effective communication.

Furthermore, it was observed that the ability of CureApp HT to improve patients’ knowledge of their disease and their motivation also helped physicians recognize changes in patients’ stages of behavioral change. When the application was started, information was provided in the form of a dialogue with the application’s characters over 14 days. This includes information on hypertension, purpose of treatment, and modification of lifestyle habits. Subsequently, the application presents specific behavioral goals tailored to each patient’s preferences and lifestyle patterns, and the patient begins a period of behavioral change. Until their next medical checkup, the patient measures their blood pressure daily, implements the knowledge they have acquired into practice to improve their lifestyle, and receives feedback from the application. During this process, patients may have specific questions or issues that they discuss with their physician during their next medical checkup. Through these interactions, physicians can recognize that the patient is in the “action” stage of the behavioral change model. In the Stage of change in the Transtheoretical Model, individuals progress through five stages: “Precontemplation,” “Contemplation,” “Preparation,” “Action,” and “Maintenance.” The “Preparation” stage is when a person is considering starting to change their behavior within a month, and the “Action” stage is when they actually change their behavior for 6 months or less, although the change has not yet become fully established [11].

Another important finding of this study was that physicians set stricter target blood pressure values. This can be understood as the first step towards improving clinical inertia. Clinical inertia in the treatment of hypertension is defined as “not starting treatment despite having hypertension or not intensifying treatment despite not achieving the target blood pressure indicated in the guidelines” [1]. The study results suggest that confirming the patient was in the “Action” stage and experiencing effective communication during the consultation made it easier for physicians to set stricter targets. Notably, this adjustment was not initiated solely by a physician but was a change in response to changes observed on the patient’s side.

Factors related to physicians, patients, and the healthcare system are believed to contribute to clinical inertia. Physicians-related factors include managing a large number of patients, inability to spend enough time with patients, and reactive interventions. Patient-related factors include low health literacy and a lack of communication between physicians and patients [7]. In our study, although there was no change in the content of what physicians communicated during consultations, physicians observed changes in what the patients said. Although physicians’ knowledge and ability to make appropriate treatment decisions are crucial in addressing clinical inertia, this study indicated that providing sufficient knowledge to the patient, improving their sense of self-efficacy through behavioral change practices, and fostering changes in patient engagement and behavioral change stage could also significantly impact resolving clinical inertia.

CureApp HT can support the realization of “concordance medicine.” “Adherence” refers to the willingness of patients to voluntarily and actively engage in treatment; however, “concordance” goes one step further and means that patients make decisions as part of a team that includes healthcare professionals. The 2019 Hypertension Treatment Guidelines [1] emphasize that achieving “concordant medicine” involves providing sufficient information and engaging in discussions about treatment plans on an equal footing with patients. The effective communication experience gained in this study can support decision-making in hypertension treatment.

Overall, our findings indicate that CureApp HT can manifest a robust antihypertensive effect by improving physician-patient communication, thereby improving patient engagement and adherence [12] and enabling physicians to set a stricter target blood pressure. Although CureApp HT was only available for 6 months at the time this study was conducted, it would be desirable to continue beyond 6 months, given the effect of CureApp HT in maintaining high patient engagement with hypertension treatment. Therefore, it remains to be explored how effective it will be when used beyond 6 months.

This study has some limitations. First, this was a recall-type questionnaire survey, and the proportion of physicians who agreed to participate and responded to all three questionnaires sent to them was relatively low. However, despite the risk of bias due to the study design and sample size, the results were consistent across multiple survey items, suggesting that the findings are of a certain significance. Second, the questions used in this study were created specifically for this research and not validated. The questions were created in line with the development intentions of the application, and we believe that they reflect the quality of physician-patient communication to a certain extent. Third, this study investigated only physicians and was not able to examine differences between individual patients. The effects discussed here may be influenced by the socio-psychological characteristics of patients, thus such research needs to be conducted with larger sample size using real-world data.

Conclusively, physicians who implemented the CureApp HT in their clinical practice experienced more effective communication through improvements in patients’ knowledge of the disease and their attitudes towards treatment. The changes facilitated by CureApp HT in patients led physicians to recognize changes in the patients’ stages of behavioral change, prompting actions, such as setting stricter blood pressure targets. Improving physician-patient communication through the app to assist in the treatment of hypertension is thought to be useful as a solution to clinical inertia.

## Supplementary information

Supplementary Figure 1

Supplementary Figure 2

Supplementary Figure 3

Supplementary Figure 4

## References

[1] Umemura S, Arima H, Arima S, Asayama K, Dohi Y, Hirooka Y, et al. The Japanese Society of Hypertension guidelines for the management of hypertension (JSH 2019). Hypertens Res. 2019;42:1235–481. 31375757 10.1038/s41440-019-0284-9

[2] Nakano M, Eguchi K, Sato T, Onoguchi A, Hoshide S, Kario K. Effect of intensive salt-restriction education on clinic, home, and ambulatory blood pressure levels in treated hypertensive patients during a 3-month education period. J Clin Hypertens. 2016;18:385–92. 10.1111/jch.12770 PMC8031489 26732187

[3] Kario K, Nomura A, Harada N, Okura A, Nakagawa K, Tanigawa T, et al. Efficacy of a digital therapeutics system in the management of essential hypertension: the HERB-DH1 pivotal trial. Eur Heart J. 2021;42:4111–22. 34455443 10.1093/eurheartj/ehab559 PMC8530534

[4] Yasutake K, Miyoshi E, Kajiyama T, Umeki Y, Misumi Y, Horita N, et al. Comparison of a salt check sheet with 24-h urinary salt excretion measurement in local residents. Hypertens Res. 2016;39:879–85. 27383507 10.1038/hr.2016.79

[5] Nomura K, Azuma H, Uetani T, Kajino S, Okazaki O, Takamizawa T, et al. Prescription status survey of digital therapeutics for hypertension: a retrospective observational study based on the matching of app data and medical records data (in Japanese). Ther Res. 2024;45:195–204.

[6] Phillips LS, Branch WT, Cook CB, Doyle JP, El-Kebbi IM, Gallina DL, et al. Clinical inertia. Ann Intern Med. 2001;135:825–34. 11694107 10.7326/0003-4819-135-9-200111060-00012

[7] Reach G, Pechtner V, Gentilella R, Corcos A, Ceriello A. Clinical inertia and its impact on treatment intensification in people with type 2 diabetes mellitus. Diabetes Metab. 2017;43:501–11. 28754263 10.1016/j.diabet.2017.06.003

[8] Hisaki F, Aga M, Tomitani N, Okawara Y, Harada N, Kario K. Daily self-reported behavioural efficacy records on hypertension digital therapeutics as digital metrics associated with the reduction in morning home blood pressure: post-hoc analysis of HERB-DH1 trial. Hypertens Res. 2024;47:120–7. 37717116 10.1038/s41440-023-01434-4

[9] Chen AM, Yehle KS, Albert NM, Ferraro KF, Mason HL, Murawski MM, et al. Relationships between health literacy and heart failure knowledge, self-efficacy, and self-care adherence. Res Soc Adm Pharm. 2014;10:378–86. 10.1016/j.sapharm.2013.07.001 PMC3923851 23953756

[10] Nishigaki N, Shimasaki Y, Yoshida T, Hasebe N. Physician and patient perspectives on hypertension management and factors associated with lifestyle modifications in Japan: results from an online survey. Hypertens Res. 2020;43:450–62. 31996815 10.1038/s41440-020-0398-0 PMC8076050

[11] Prochaska JO, Velicer WF. The transtheoretical model of health behavior change. Am J Health Promot. 1997;12:38–48. 10170434 10.4278/0890-1171-12.1.38

[12] Zolnierek KB, Dimatteo MR. Physician communication and patient adherence to treatment: a meta-analysis. Med Care. 2009;47:826–34. 19584762 10.1097/MLR.0b013e31819a5acc PMC2728700

