# Supplementary material for: Digital therapeutic for hypertension improves physician-patient communication and clinical inertia: a survey of physicians who implemented CureApp HT in clinical practice
Source: Hypertens Res. 2024 Oct 12;48(2):470–7. doi: 10.1038/s41440-024-01899-x (PMC11794138; doi:10.1038/s41440-024-01899-x)
Supplement: Supplementary file 1 — Supplementary Figure 1 [file 41440_2024_1899_MOESM1_ESM.pdf]

Supplementary Figure 1

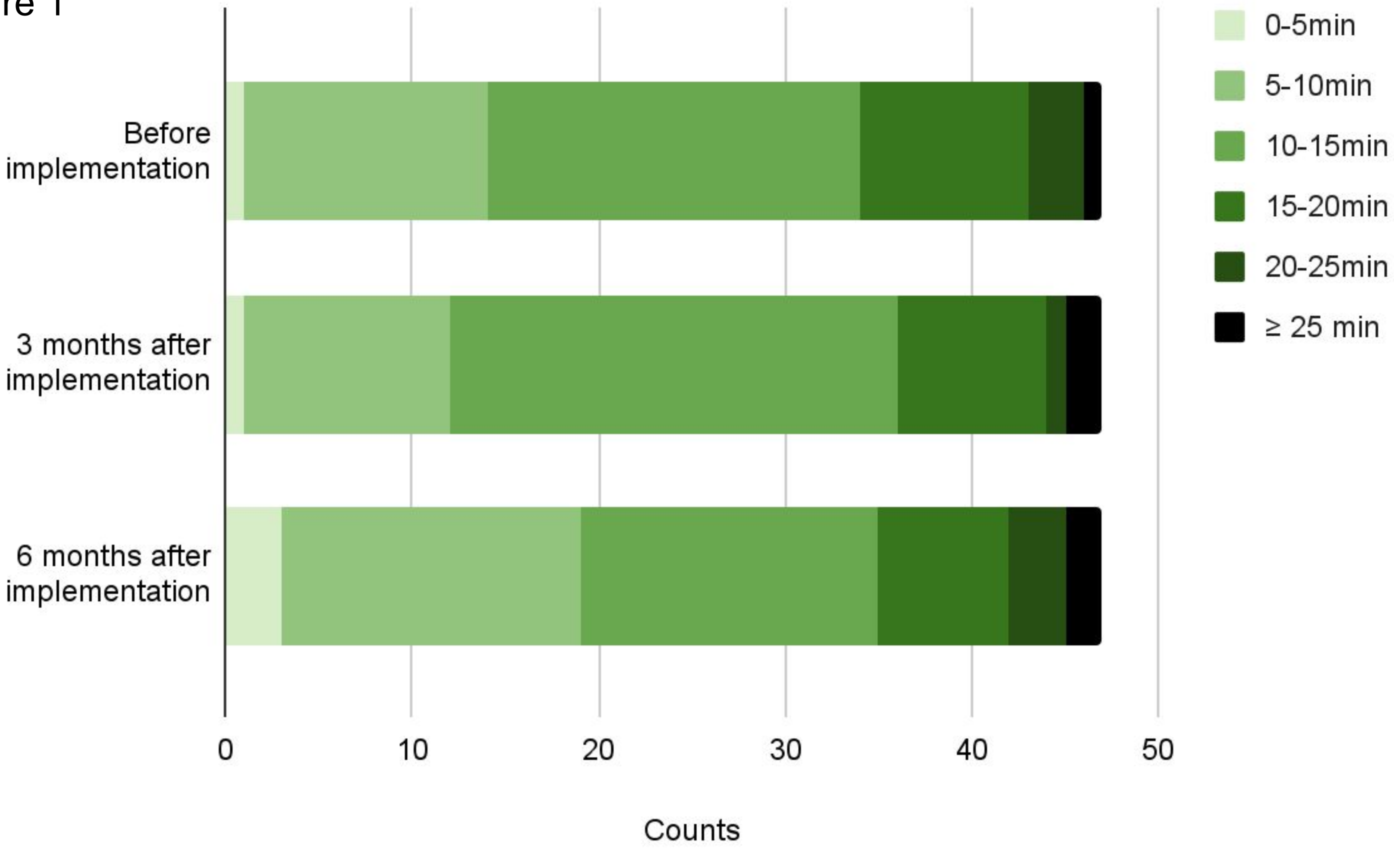

The length of consultation time spent with patients during their initial visit at the baseline and at 3 and 6 months after implementing CureApp HT.
