# Supplementary material for: Digital therapeutic for hypertension improves physician-patient communication and clinical inertia: a survey of physicians who implemented CureApp HT in clinical practice
Source: Hypertens Res. 2024 Oct 12;48(2):470–7. doi: 10.1038/s41440-024-01899-x (PMC11794138; doi:10.1038/s41440-024-01899-x)
Supplement: Supplementary file 4 — Supplementary Figure 4 [file 41440_2024_1899_MOESM4_ESM.pdf]

Supplementary Figure 4

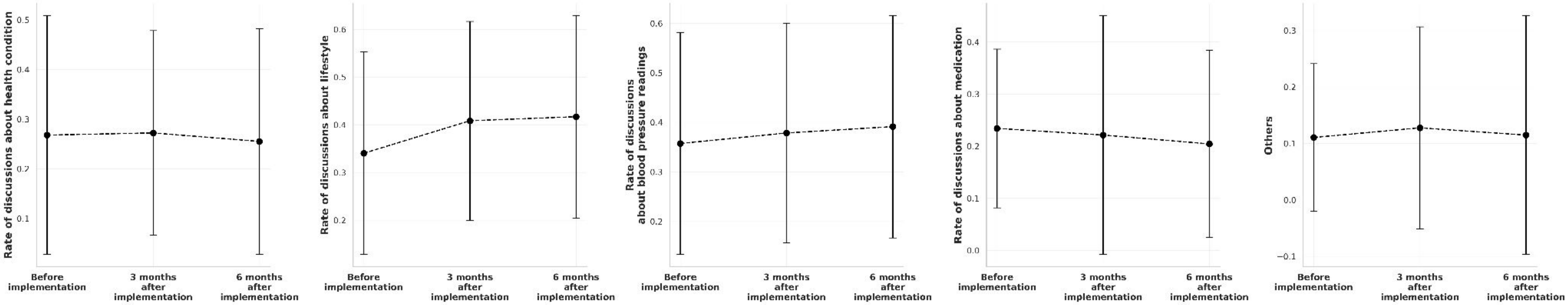

The proportion of topics discussed by physicians at the baseline and after 3 and 6 months of implementing CureApp HT (error bars represent standard deviation). *p<0.05
